# Optimizing Hysterectomy: A Prospective Comparative Analysis of Surgical Techniques and Their Impact on Women’s Lives

**DOI:** 10.3390/jpm14030265

**Published:** 2024-02-29

**Authors:** Aslihan Yurtkal, Mujde Canday

**Affiliations:** Department of Obstetrics and Gynecology, Faculty of Medicine, Kafkas University, 36000 Kars, Turkey; mujde.canday@kafkas.edu.tr

**Keywords:** hysterectomy, quality of life, female sexual function, anxiety scale, patient satisfaction, minimally invasive surgery, surgical techniques, pain assessment, post-operative recovery, women well-being

## Abstract

Study Objective: To investigate diverse hysterectomy techniques to determine their influence on patient outcomes, including pain levels, sexual function, anxiety, and quality of life. Of particular focus is the comparison between vessel sealing and traditional suturing in abdominal, vaginal, and laparoscopic hysterectomies. This study is unique in its comprehensive evaluation, considering patient satisfaction, recommendation rates, recovery times, and various other aspects. Method: Our prospective cohort study adhered to ethical guidelines, involving a meticulous assessment of patients, including medical history, anxiety levels, pelvic pain, sexual function, and quality of life. Surgical methods were explained to patients, allowing them to actively participate in the decision-making process. Sociodemographic information was collected, and exclusion criteria were applied. Hysterectomy methods included total abdominal hysterectomy (TAH), laparoscopic hysterectomy (TLH), vaginal hysterectomy (VH), and a modified vaginal technique known as VH Mujas. Several parameters were recorded, including operation indications, uterine volume, hospital stay, operation duration, pre-operative and post-operative complications, and more. Results: In all groups, a statistically significant increase was found in pre-operative–post-operative FSFI sexual function values (*p* < 0.001). The patient’s basal Beck Anxiety Scale scores significantly decreased following the decision for vaginal surgery, both in the VH and VH Mujas groups (*p* < 0.05). However, Beck Anxiety Scale scores at patients’ initial assessments significantly increased following the decision for abdominal and laparoscopic surgery (*p* < 0.001). According to the results of the SF-36 quality of life assessment, an increase was observed in all post-operative quality of life parameters in patients who underwent surgery with different methods due to VH (*p* < 0.05). Conclusions: Our comprehensive comparison of hysterectomy techniques demonstrated that VH, particularly when utilizing the Mujas technique, outperforms other hysterectomy methods regarding patient safety and post-operative satisfaction but also offers the benefit of minimal invasiveness. Notably, this is reflected in improved quality of life, enhanced sexual function, lower pain scores, and favorable cosmetic results. The success of a hysterectomy procedure depends on precise indications, surgical planning, proper patient selection, and effective communication. This study emphasizes the significance of these factors in achieving optimal outcomes. The development of specialized vascular closure devices can further enhance the feasibility of vaginal hysterectomy, making it a preferable choice in gynecological surgery. The study contributes valuable insights into selecting the most suitable hysterectomy method for patients and optimizing their recovery.

## 1. Introduction

Hysterectomy is one of the most commonly performed surgeries in gynecology practice [1]. The majority of hysterectomies are performed for benign indications [2]. It is estimated that one in every three women will undergo a hysterectomy at some point in her life [3].

Numerous symptoms that appear before hysterectomy, such as heavy and prolonged menstrual bleeding, chronic pelvic pain, dyspareunia, dysmenorrhea, and cosmetic results of prolapse, can have a negative impact on the quality of life and sexual function [4]. A hysterectomy performed to relieve these symptoms can also be expected to have positive effects on the patient’s quality of life and sexual function [5]. 

Currently, there is no consensus on how minimally invasive hysterectomy techniques affect female sexual function [6]. A common concern voiced by women during hysterectomy counseling is the potential impact of the procedure on sexual function [7]. Notably, during counseling for hysterectomy, some women express the belief that the procedure will improve sexual function or remove barriers to sexual enjoyment. In contrast, others are concerned that hysterectomy will diminish their sexual function [8]. At the same time, some have serious concerns about their sexual function in the post-operative period, leading to increased pre-operative stress. Additionally, the severity and/or chronicity of the symptoms leading to the decision for hysterectomy and its impact on daily life and quality of life can also create stress for the patient. The choice of surgical method can further contribute to pre-operative stress.

Considering the relevance and importance of this operation, it is crucial to continue improving surgical techniques to ensure that women requiring a hysterectomy can benefit from the best and least invasive operative methods available. The main purpose of hysterectomy is to improve the patient’s quality of life by relieving symptoms. Therefore, careful evaluation of potential side effects is crucial. Providing counseling services to patients and detailing the surgical methods are essential in selecting the ideal approach and optimizing outcomes. In this regard, our study aims to compare vessel sealing and conventional suture techniques in abdominal and vaginal hysterectomy, along with laparoscopic hysterectomy, considering pain scores, sexual function assessment, anxiety scale, and quality of life scale. Our study is the first to evaluate hysterectomy methods comprehensively using all these parameters. To determine the most suitable method for patients, we aim to elucidate the details of hysterectomy techniques and review the advantages and disadvantages of different approaches. This investigation also encompasses hospital stay duration, need for readmission, bleeding volume, pre-operative and post-operative complications, operation duration, patient satisfaction, recommendation rate, and recovery time. Furthermore, we have evaluated the time to return to daily routines. This study was conducted to identify the most appropriate hysterectomy technique for patients and optimize their recovery period.

## 2. Material and Methods

This study was planned for a prospective cohort and conducted according to the principles of the Declaration of Helsinki. The ethics committee approval for the study was obtained from the Clinical Research Ethics Committee of Kafkas University with decision number 80576354-050-99/210.

### 2.1. Patient Selection, Examination, and Questionnaires

Detailed medical histories and examinations were conducted on patients at the gynecology outpatient clinic. Findings and treatment options were thoroughly explained, and patient inquiries were comprehensively addressed. The same team of physicians carried out all examinations and consultations. Prior to discussing treatment options, patients’ baseline anxiety levels were assessed using the Beck Anxiety Scale (BAS). Additionally, during the initial consultation, pelvic pain was evaluated using the Visual Analog Scale (VAS), sexual function was assessed with the Female Sexual Function Index (FSFI), and quality of life (QoL) was measured with the Short Form-36 (SF-36). The surveys were administered in face-to-face sessions under the physician’s observation. Sociodemographic information was documented. In the second consultation, after evaluating pre-operative test results, medical or surgical (uterus-preserving or hysterectomy) treatment options were presented based on the findings.

The final treatment choice was determined through collaborative decision-making with active patient participation. Patients who opted for medical treatment were not included in the study. Patients recommended for endometrial ablation, premenopausal women requiring oophorectomy as part of their surgery, those with suspected malignancy, illiteracy, or individuals unable to respond to questionnaire questions due to mental or psychological limitations were considered exclusion criteria. Patients who decided to undergo hysterectomy were provided with detailed explanations of all surgical methods, complete with illustrations. The choice of the most suitable surgical method was aimed at aligning with the patient’s expectations. The potential complications associated with each surgical approach were thoroughly discussed. The final surgical method was determined through consensus between the physician and the patient. Following the decision on the surgical method, the BAS was administered a second time to assess changes in anxiety levels related to the chosen treatment method and to identify any differences in anxiety levels due to the selected surgical approach. Pre-operative anesthesia evaluations were performed, and recommendations were followed. No pre-operative analgesic treatments were administered to any patient. All patients were advised to wear knee-high compression stockings. All patients in the study voluntarily participated, having signed a detailed consent form outlining the study’s specifics.

### 2.2. Patient Parameters

The age, prior abdominal surgeries, education status, parity information, systemic illness inquiries, smoking habits, and body mass index (BMI) information were recorded.

### 2.3. Surgical Methods

The same team of physicians performed all surgeries. We presented five surgical method options and excluded approaches that combined abdominal and vaginal surgery or those requiring additional equipment like V-NOTES. Robotic surgery was unavailable, and urinary incontinence surgeries were not performed on any patients. Cuff closures for all patients were carried out using a single-layer continuous no. 1.0 Vicryl Ethicon suture, while the subcutaneous skin of all patients was closed using no. 3.0 rapid Vicryl. Our operations aimed to standardize the materials and instruments used. Given that LigaSure is a safe and efficient instrument for laparoscopic hysterectomy, we used it as the bipolar vessel sealing system (BVSS) [9].

Total Abdominal Hysterectomy (TAH) 1: Conventional abdominal hysterectomy was performed using a Heany-type clamp and a 1.0 Vicryl Ethicon suture ligature. A uterosacral ligament shortening operation was performed when deemed necessary.

TAH 2: Operations performed using the BVSS (only LigaSure™, Medtronic/Covidien, Minneapolis, MN, USA) were used. Sutures were used only for cuff and skin closure.

Total Laparoscopic Hysterectomy (TLH): A history of multiple abdominal surgeries was not considered a contraindication for TLH. TLH was not recommended for cases with a bulky uterus, virgins, a history of umbilical hernia repair involving mesh application, or a cervix that had been previously removed.

The surgical steps followed a conventional approach. All cases were performed using the same brand of laparoscopy tower. In operations, only LigaSure™ Maryland and RUMI manipulators were utilized. A uterosacral ligament shortening operation was performed when deemed necessary.

Vaginal Hysterectomy (VH): The surgical steps followed a conventional approach. Hydrodissection was performed using a solution prepared with 100 cc of saline, 1 ampule of lidocaine, and 1 ampule of epinephrine. All patients received vaginal tampons post-operatively. An immobile uterus was considered an exclusion criterion for VH. Prolapse degrees were not taken into account. An increased number of prior abdominal surgeries was not considered a contraindication for VH. While McCall Kuldoplasty was applied to all patients, sacrospinous fixation was added when necessary.

VH Mujas: In all modified stages of vaginal hysterectomy, LigaSure was utilized. This technique, referred to as “Mujas”, involved using an 18.8 cm small-jawed open sealer capable of sealing vessels up to 7 mm in diameter [10]. A hydrodissection was performed. After securing the cervix with a single-toothed clamp, traction was applied. An incision was made on the anterior vaginal wall in a reversed V-shape to remove a triangular piece of tissue, improving the surgical field visualization during paracervical dissection and obviating the need for final anterior colporrhaphy stages. The apex of the reversed V incision was adjusted based on the potential need for anterior colporrhaphy. The triangular bases extended to the corners of the cervix, and the anterior and posterior cervix were prepared for dissection with a circular incision. After dissecting the anterior vaginal tissue, paracervical planes were accessed using the small-jawed vessel sealer (thyroid LigaSure). The uterus was consistently lateralized in the opposite direction, requiring proper traction for optimal exposure. Effective dissection, facilitated by a blunt-tipped vessel sealer, was essential, especially at the uterine artery and its branches. In cases requiring oophorectomy, the ovaries were held with Babcock forceps, and the infundibulopelvic ligament was coagulated and cut using a blunt-tipped open vessel sealer. For salpingectomy, a small-jawed vessel sealer was used. No sutures were used until the uterus and/or tubal ovarian structures were removed as a single piece. McCall kuldoplasty was performed on all patients, and sacrospinous fixation was added in cases of total prolapse. The cuff was continuously closed using a single layer of a 1.0 Vicryl suture, and all patients received post-operative vaginal tampons.

The inclusion and exclusion criteria considered in the selection of surgical methods are expressed in Table 1.

In selecting the surgical method, factors such as parity, the presence of systemic disease, and the number of previous abdominal surgeries were not considered. Our patients were presented with all possible surgical alternatives, taking into account the indications and contraindications. Except for those patients for whom only one surgical option remained after considering the inclusion and exclusion criteria, all others were provided with detailed explanations of alternative methods and the surgical process, both verbally and in written form, to aid in making the final decision on the surgical approach.

### 2.4. Operation Evaluation

The chosen surgical method, operation indications, pathology reports, uterus volumes (axbxc), length of hospital stay (days), need for readmission, pre-operative and post-operative 6-hour hemogram/hematocrit values, and perioperative complications were recorded. 

Operation Duration: The operation duration was defined as follows: for TAH and VH, it started with the first incision, and for TLH, it began with the insertion of the first trocar and ended with cuff closure. This definition was chosen because of the variable composition and experience levels of operating room personnel, particularly in vaginal operations, where suspension surgery durations can vary. The study was conducted at a university hospital, and different skin closure times were observed due to student training sessions. Anesthesia administration and awakening times were not included in the operation duration to avoid the influence of multifactorial patient-dependent variables.

Anesthesia and perioperative pain control: The Enhanced Recovery After Surgery (ERAS) guideline [11] was considered within the conventional rules.

### 2.5. Post-Operative Evaluation

Patients had scheduled follow-up appointments on the 10th day and the first and sixth months after discharge. The same physicians consistently performed these follow-up assessments. On the 10th day post-operatively, a VAS inquiry was conducted before the examination. A thorough examination of early post-operative complications, patient satisfaction using a Likert scale, willingness to recommend the operation, and return to daily routine were assessed. Detailed examinations were performed at the 6-month follow-up appointments to evaluate potential late-term complications. Additionally, patients completed the FSFI and SF-36 questionnaires.

### 2.6. Statistical Analysis

The conformity of the continuous variables in the study to the normal distribution was evaluated graphically and by the Shapiro-Wilks test. It was determined that none of the continuous variables fit the normal distribution. The mean ± SD (standard deviation) and median (minimum–maximum) values were given in the descriptive statistics of the variables.

Cross-tabulations were created to compare categorical variables according to hysterectomy type, and number (*n*), percentage (%), and chi-square test statistics were given.

Kruskal–Wallis’s non-parametric analysis of variance was used to compare age, uterine volume, number of previous abdominal surgeries, length of hospital stays, and time to hysterectomy according to hysterectomy type. In addition, Kruskal–Wallis’s non-parametric analysis of variance was used to compare pre-op and post-op values according to hysterectomy type. Bonferroni correction was made for pairwise comparisons, and analysis results were given. 

A dependent sample Friedman’s test was used to compare VAS values at measurement times (pre, day 1, and day 10). The Wilcoxon signed rank test was used to compare the pre-op–post-op values of Hg, FSFI, and SF36 and two pre-operative assessments of BAI.

IBM SPSS Statistics 21.0 (IBM Corp., released in 2012). IBM SPSS Statistics for Windows, Version 21.0. Armonk, NY, USA: IBM Corp.) and MS-Excel 2007 programs were used. The statistical significance level was accepted as *p* < 0.05.

## 3. Results

The sociodemographic characteristics of our study group are shown in Table 2. Among the indications of the hysterectomy procedure, uterine fibroids, adenomyosis, and POP were the most common (Table 2).

According to hysterectomy type classification, a statistically significant difference was found between the age values of the individuals according to hysterectomy type (*p* < 0.001), in the distribution of educational level (*p* = 0.002), parity (*p* = 0.008), and systemic diseases (*p* = 0.010). At the same time, there was no statistically significant difference in habit distribution (*p* > 0.05) (Table 3). 

According to the data shown in Table 4, a statistically significant difference was found between the vaginal route and the other techniques in terms of age (TAH 2-VH Mujas; *p* = 0.001, TAH 2-VH; *p* < 0.001, TLH-VH; *p* = 0.009). 

Comparing the groups in terms of uterine volume value, a statistically significant difference was found between abdominal hysterectomy and other techniques (TAH 2-VH Mujas; *p* = 0.021, TAH 2-VH; *p* = 0.002, TLH-TAH; *p* = 0.025, TAH-VH Mujas; *p* = 0.001, TAH-VH; *p* = 0.007). 

There was a statistically significant difference between TAH 2-TLH, TAH 2-VH, and TLH-TAH in the pairwise comparison of the groups regarding previous abdominal surgery value (*p* = 0.006, *p* = 0.034, *p* = 0.049).

A statistically significant difference was found between TAH 2-TLH, TAH 2-VH Mujas, TAH 2-VH Mujas, TAH 2-VH, TLH-TAH, TLH-VH Mujas, TAH-VH Mujas, TAH-VH, and VH Mujas-VH (*p* = 0.015, *p* < 0.001, *p* = 0.047, *p* = 0.001, *p* = 0.001, *p* < 0.001, *p* < 0.001, *p* = 0.004, *p* < 0.001) in terms of hospital stay. 

A statistically significant difference was found between TAH 2-TLH, TAH 2-TAH, TAH 2-VH Mujas, TLH-VH Mujas, TAH-VH Mujas, TAH-VH, and VH Mujas-VH in terms of operation times (*p* < 0.001, *p* < 0.001, *p* < 0.001, *p* < 0.001, *p* < 0.001, *p* < 0.001, *p* = 0.008, *p* < 0.001).

Significant differences in post-operative Hg levels were observed among hysterectomy-type groups (*p* < 0.001), most significantly in the TAH group (Table 4). Significant differences also appeared in pre-operative vs. post-operative Hg values within the TAH 2, TLH, VH, and VH Mujas groups (*p* < 0.001). Statistically significant differences were found when comparing post-operative Hg levels among specific groups, such as TAH 2-TAH, TLH-TAH, TLH-VH Mujas, TAH-VH Mujas, TAH-VH, and TAH-VH (*p* < 0.001, *p* = 0.008, *p* = 0.001, *p* < 0.001, *p* = 0.001) (Table 5).

Hysterectomy-type groups observed significant differences regarding pre-operative, post-operative first-day, and tenth-day VAS measurements. In all groups, the VAS value increased on post-op day 1 when compared to the pre-operative value and decreased on day 10 compared to day 1 (*p* < 0.001) (Table 6). FSFI values increased over time in all groups, and there was a statistically significant difference among the hysterectomy types in terms of pre-operative and post-operative FSFI measurements (*p* < 0.001) (Table 6).The findings in Table 6 have been interpreted. A statistically significant difference was observed among the hysterectomy-type groups regarding BAS measurements (*p* < 0.001). The highest BAS score in the second interview was found in the TAH group, while the lowest was in the VH Mujas group. Additionally, there was a significant difference between the two time-dependent measurements of BAI values of individuals in the operation groups (*p* < 0.001). In the TAH, TAH 2, and TLH groups, BAS scores increased over time but decreased in the VH and VH Mujas groups.

Pre-operative SF36 energy/vitality measurements were similar among hysterectomy groups (*p* > 0.05), but other SF36 measurements showed significant differences (*p* < 0.05).

Post-operative SF36 measurements revealed significant differences in physical functioning (PF), role limitations due to physical problems (PR), role limitations due to emotional problems (ER), energy/vitality (EVT), mental health (MH), bodily pain (BP), and general health (GH) perception among hysterectomy groups (*p* < 0.05). Social functioning measurements were similar among groups (*p* > 0.05) (Table 6).

A significant difference was found in the two time-dependent measurements (pre-op and post-op) of SF36 PF, PR, ER, EVT, MH, SF, BP, and GH perception in all hysterectomy groups (*p* < 0.001). In all groups, SF36 values increased over time (Table 6).

The findings in Table 7 have been interpreted. Statistically significant differences were found between various groups in pairwise comparisons of VAS pre-op, day 1, and 10th-day measurement values. For VAS pre-op, significant differences were observed in comparisons between TAH 2-TLH, TAH 2-TAH, TAH 2-TAH, TAH 2-VH Mujas, TAH 2-VH, TLH-VH Mujas, TLH-VH, TAH-VH Mujas, and TAH-VH (*p* = 0.001, *p* = 0.029, *p* = 0.002, *p* = 0.016, *p* < 0.001). For VAS day 1, significant differences were observed in comparisons between TAH 2-TAH, TAH 2-VH Mujas, TLH-TAH, TLH-VH Mujas, TLH-VH, TAH-VH Mujas, TAH-VH, and VH Mujas-VH (*p* < 0.05). For VAS 10th day, significant differences were observed in comparisons between TAH2-TAH, TAH2-VH Mujas, TLH-TAH, TLH-VH Mujas, TAH-VH Mujas, TAH-VH Mujas, and TAH-VH (*p* < 0.001).

In the pairwise comparison of the groups, a statistically significant difference in BAI first interview measurement value was only found between TAH2-VH (*p* = 0.006). Significant differences were found in the BAI second interview measurement value between TAH 2-TAH, TAH 2-VH Mujas, TLH-TAH, TLH-VH Mujas, TAH-VH Mujas, TAH-VH Mujas, TAH-VH, and VH Mujas-VH (*p* = 0.003, *p* < 0.001). Additional details of pairwise comparisons for other variables are presented in Table 7.

Our complication rates were within the expected range. There were no fatalities or requirements for blood transfusions. In two cases, we had to transition from laparoscopic to open surgery. One case was identified as malignant, originating from the intestines, and in the other case, dense adhesions from previous abdominal surgeries made abdominal access impossible.

## 4. Discussion

Hysterectomies, commonly performed for non-malignant reasons, can be carried out through various approaches, including abdominal, vaginal, and laparoscopic methods. The choice of approach is influenced by factors such as the size of the uterus and vagina, the presence of extrauterine disease, ease of access to the uterus, the need for concurrent procedures, the surgeon’s experience, whether the procedure is planned or urgent, the hospital’s technologic equipment and the level of support available, and the informed preferences of the patient. Vaginal and laparoscopic approaches are considered minimally invasive compared to abdominal hysterectomy, offering shorter hospitalization and recovery times. The pursuit of minimally invasive but highly efficient methods demonstrates a dedication to enhancing patient results and accelerating the healing process [12].

ACOG recommends minimally invasive approaches, prioritizing vaginal hysterectomy [13]. Although advancements in surgery have led to an increase in minimally invasive surgery [14], abdominal hysterectomy remains a viable option for some patients [15]. Surgeons often opt for the abdominal route because they believe it provides more space to access vascular pedicles, which boosts their confidence [16]. Safe hemostasis has always been a main topic in surgery [17]. Surgical advancements have introduced energy-based vessel sealing (EBVS) devices, reducing operative duration and blood loss compared to traditional suture ligation techniques [18]. 

In 2001, LigaSure, a novel surgical device for sealing vessels during surgeries, was introduced [19]. It effectively seals vessels by melting collagen and elastin in the tissue, permanently closing vessels with a diameter of up to 7 mm in approximately 5 s [20]. McLellan et al. reported their experience of performing abdominal hysterectomies using bipolar coagulation (LigaSure^®^) [21]. The abdominal approach provides the surgeon with an excellent view of the surgical site, allowing the removal of uteri of any size. However, this technique has drawbacks, such as extensive incisions, potential wound-healing complications, and longer recovery periods. Although laparoscopic hysterectomy offers advantages over the abdominal approach, it comes with longer operative times [22], which is linked to adverse outcomes irrespective of the surgical method used [23]. Compared to the abdominal approach, minimally invasive surgery (MIS) has the shortest recovery, lowest post-operative pain levels, and shortest hospital stays [24]. In some clinics, hysterectomies are performed on an outpatient basis. When we evaluate MIS techniques internally, we observe the vaginal hysterectomy to be the most cost-effective and the laparoscopic approach to be the most expensive one [25]. Laparoscopy employs coagulation for hemostasis, while traditional abdominal and vaginal procedures use sutures. Incorporating the principles of laparoscopic hemostasis into vaginal hysterectomy aims to combine the advantages of both techniques. Vaginal hysterectomy is proven to be safe and advantageous in many respects, yet it accounts for less than 30% of all hysterectomy techniques. Reasons for the preference for alternatives to vaginal hysterectomy include inadequate training, cases with large uteruses, and nulliparity [26]. Vaginal hysterectomy was originally common for uterine prolapse but has gained broader use for cases involving fairly normal-sized uteruses [27]. It offers benefits such as less post-operative pain, satisfactory aesthetic results, a reduced incidence of post-operative fever, a lower risk of ureteral damage, shorter hospital stays, decreased hospital costs, fewer complications, and shorter recovery periods when compared with abdominal and laparoscopically assisted vaginal hysterectomy [28]. Surgeons previously avoided vaginal hysterectomies due to challenges such as limited space for sutures on vascular pedicles, potential bleeding that obstructs the surgical view, and difficulties in achieving hemostasis in a narrow space [29]. These challenges have been addressed by advanced EBVS devices, making vaginal hysterectomy safer [30]. We hypothesize that EBVS in vaginal hysterectomies will have a complication rate equal to or better than previous reports, with a primary hypothesis that urinary complications will not exceed the 0.64% rate reported in the FINHYST study [31]. We constantly seek faster and more efficient hemostatic techniques to replace traditional suture ligation [32]. A previous meta-analysis of randomized controlled trials showed a 28% reduction in mean operative time with EBVS compared to conventional surgical methods across various procedures [20]. Some authors have adopted EBVS instruments in vaginal hysterectomies with success rates of 60% to 100% [33]. Our results were consistent with the literature regarding the duration of operative techniques favoring VH. EBVS instruments require less space during vaginal hysterectomies [34] and have been successfully used for several years [35]. This success can be attributed to the use of fewer instruments simultaneously, preventing excessive traction on vascular pedicles. Based on our experience, performing concurrent oophorectomies when necessary and ligating the lateral parts of large uteruses, where placing hemostatic clamps and implementing a safe suture are challenging, makes BVS more feasible. Previous randomized controlled trials have demonstrated the safety and effectiveness of VH with the BVS technique [36]. This aligns with existing literature [30]. Traction of tissue during vaginal hysterectomy for better visualization can lead to increased post-operative pain and potential nerve damage, resulting in a higher incidence of post-operative urinary symptoms [37].

We believe that the preference for costly, high-tech, and not easily accessible techniques such as single-site LS, V-notes, and especially robotic surgery in benign pathologies is a subject of debate. For V-NOTES, additional attachments are required, increasing costs and prolonging the operation time. Moreover, there is not enough strong evidence to recommend using NOTES over conventional multiport laparoscopic surgery (MLS) [38].

The time required for TLH setup is also long, and in the meantime, experienced and trained operating room staff assistance is needed, and the learning curve of the technique is rather steep for the surgeons. VH is easier and faster when no additional experienced assistance is needed. In addition, minimally invasive techniques reduce surgical trauma, which aids in post-operative recovery; clinicians are investigating various minimally invasive strategies alongside traditional laparoscopy [39]. With this exploration, we developed our method with the Mujas technique, and the likelihood of bladder injury is also reduced. To maintain hemostasis when you prefer BVS for ligating vessels, we observed less post-operative pain and a quicker recovery, especially compared to other techniques. There were no issues related to visualization in the case of oophorectomy necessity. Ligating the ovarian artery with LigaSure was much safer, more comfortable, and faster. Moreover, placing sutures on the ovarian artery is much riskier, or the knot may loosen [40]. In our case series, we have not noticed a higher rate of complications associated with BVS usage, except for one case where a vulvar skin burn was observed as a complication. Although it was feasible to discharge patients on the same day as the surgery, the rural setting of our location and the predominantly lower socioeconomic status of our patient population made same-day discharges impractical due to social factors. We stress the significance of embracing vaginal techniques, as not all hospitals may have access to an adequate number of skilled operating room staff and expensive endoscopic surgical equipment required for ensuring safe working conditions. While patients who undergo various hysterectomy techniques have reported satisfactory short-term quality-of-life outcomes, endorsing vaginal hysterectomy as the preferred and recommended primary technique, it is essential to note that definitive conclusions would necessitate long-term quality-of-life surveys.

Sexual health is a vital aspect of QoL and can have a significant impact, even on older people [41]. Conditions like abnormal uterine bleeding (AUB), chronic pelvic pain, and pelvic organ prolapse (POP) often lead to a decrease in women’s libido, causing them to avoid sexual activity and seek medical assistance. Psychological, physiological, and social factors influence female sexual function. It is more connected to a woman’s self-perceived body image than physical changes resulting from POP [42]. Women with POP often restrict their sexual activity due to concerns about their perceived attractiveness and the fear of incontinence, which can lead to discomfort, decreased sexual excitement, and ultimately result in abstaining from sexual activity [43]. Pelvic floor dysfunction (PFD) tends to increase with age and can have adverse effects on sexual activities [44]. Sexual dysfunction is estimated to affect approximately 30% to 50% of the general population, but in women with PFD, the reported incidence increases significantly to approximately 50% to 83%. The primary reasons identified for the decline in a woman’s sexual well-being include concerns about their vaginal appearance among those with pelvic organ prolapse, dyspareunia, and coital incontinence in women dealing with urinary incontinence, and the apprehension of experiencing fecal incontinence-related issues [45].

Hysterectomy is a common major gynecological procedure, with 85% of patients undergoing it being sexually active [46]; therefore, it is crucial to consider its impact on sexual life and the pre-operative anxiety and concerns of patients [7]. Regrettably, this anxiety is often unspoken by patients and not fully addressed by clinicians [5]. Reports on how hysterectomy affects sexual function have yielded conflicting results, partly due to the use of varying and often inadequate parameters to assess sexual function [5]. The evaluation of sexual activity and function should be an integral part of urogynecology assessments, as this information is crucial for therapy planning [47]. A new model of the ‘sexual response cycle’ that encompasses physical, emotional, and cognitive aspects helps in understanding sexual difficulties before and after hysterectomy. There is insufficient evidence to support sexual dysfunction arising solely from disruptions or changes in anatomical relationships. Some studies suggest that women experience an improved quality of life and enhanced sexual function after hysterectomy, regardless of the surgical method, due to the resolution of organic issues that adversely affect sexuality [48]. The review conducted by Danesh et al. emphasizes that most of the sexual disorders improve after hysterectomy for benign uterine diseases, and it highlights that most patients who were sexually active before the surgery are experiencing the same or better sexual functioning after the surgery [49].

However, others argue the opposite, claiming that changes in pelvic anatomy, blood supply, and the loss of nerve tissue may lead to a decline in sexual function [8]. Radosa et al. compared different hysterectomy techniques and found that female sexual function improved regardless of the surgical approach [50]. According to our study results, we observed a general improvement in women’s FSFI scores after all hysterectomy techniques, with the most significant improvement seen in VH. When VH is further evaluated as two distinct groups, conventional and VH Mujas techniques, the most notable increase in FSFI values was observed in the VH Mujas group.

Numerous randomized trials have compared operative outcomes and morbidity between AH and MIS. However, only a few have assessed QoL using validated assessments, and their results have been inconclusive [51]. Some trials favor the MIS approach regarding post-operative QoL, while others find no significant difference between the AH and MIS groups [52].

On the other hand, Bartels et al. concluded in their review article that MIS hysterectomy is associated with improved QoL in the short-term post-operative phase compared to AH for benign diseases [53]. Authors assessing the effects of hysterectomy on sexual function over the past decade have reported varying outcomes, including post-operative improvement, no impact, or reduced libido. In their studies, Hoffmann et al. have suggested that hysterectomy, even when the cervix is removed for benign diseases, has positive effects on sexual function independent of surgical techniques [54]. Ayoubi et al. suggested that laparotomy procedures were associated with long-term impairment of quality of life due to increased post-operative pain levels and delayed resumption of normal daily activities compared to laparoscopy [55]. Adding oophorectomy to hysterectomy was found to reduce post-operative libido in premenopausal patients [56] significantly. Differences in the age composition of study populations also limit the generalization of post-hysterectomy quality of life and sexual function findings, as both parameters have been negatively affected by menopause [41]. In our study, the mean age of the patients was 53.73 ± 8.41. Premenopausal patients who had oophorectomy as part of their hysterectomy were deliberately excluded from our study due to the well-established knowledge of the adverse effects of surgical menopause on quality of life and sexual well-being. We believe conducting studies specifically focused on this patient subgroup would be beneficial.

The best results for anxiety were observed in VH operations, possibly due to the older age of the women undergoing VH. These patients often have chronic complaints related to pelvic organ prolapse and are eager to address this issue surgically. In our study, the assessment of anxiety was conducted to evaluate the impact of the chosen surgical method on the patient’s baseline anxiety and determine the appropriate surgical approach. We investigated the effect of detailing the surgical method to the patient, verbally and, when necessary, through illustrations, on the patient’s baseline anxiety and how the selected surgical method influenced it.

Our study found that when the surgeon thoroughly explained the surgical method to the patient, utilized visual aids when necessary, and ensured that the patient had a comprehensive understanding of the operation, it reduced the patient’s anxiety. Furthermore, the preference for MIS methods was shown to decrease the existing anxiety in patients.

Essentially, the objective here is to compare different hysterectomy methods while considering their effects on anxiety, and in this regard, there is no similar study in the literature. As the literature, specifically as highlighted in the review by Darwish et al., indicates, studies on anxiety have primarily focused on the effects of hysterectomy, and the conclusion of the study reported that hysterectomy for benign gynecological conditions is not adversely associated with anxiety [57].

Our study possesses the distinction of being the first in literature to explore a multitude of parameters. We compared hysterectomy methods with previously unexamined variables. Additionally, it is the first study to comprehensively evaluate all commonly used hysterectomy methods within such a wide spectrum. No other study in the literature evaluates all these hysterectomy methods together in such a broad scope. Furthermore, in our study, the fact that the same two surgeons performed all cases allows for a more stable assessment of the results. These parameters are the strengths of our study.

However, due to our location as a rural hospital, we may have limitations in assessing early discharge, and we may still need to be able to accurately evaluate early discharge due to transportation difficulties faced by patients traveling to the hospital. Additionally, our study did not assess hysterectomy methods in terms of cost-effectiveness. These factors can be considered limitations of our study.

## 5. Conclusions

According to our results, VH, especially with the Mujas technique, has been superior to all other routes of hysterectomy concerning patient safety and post-operative satisfaction, which was clarified by the significant improvement in QoL and FSFI scores, the lowest VAS scores, the surgical method’s minimally invasive nature, and the absence of sutures, which contribute to the best control of pre-operative anxiety and satisfying cosmetic results due to the use of natural orifices. Tailoring treatments to individual patients should be the starting point of the entire surgical process to achieve success, which is an influential and respected approach in healthcare for women’s well-being.

The most significant factors in surgical success are accurate indication, proper planning, effective communication, correct patient selection, determining the appropriate surgical method, and providing an effective field of view during the operation. Enhancing the safety and ease of performing vaginal hysterectomy has sparked a recent surge of interest in developing quicker, more straightforward, and more efficient hemostatic techniques, moving away from traditional knot tying for vessel ligation. In conclusion, developing specialized angled and flexible vascular closure devices for VH will make VH more feasible and preferable.

## Figures and Tables

**Table 1:** Inclusion and Exclusion Criteria for Selecting a Surgical Method.

Criteria	VH-VH Mujas	TAH 1-TAH 2	TLH
Inclusion Criteria			
Uterine Size	Small to moderate	Any size	Small to moderate
Indications	POP AUB resistant to medical treatment	Myoma Uteri Adenomyozis AUB resistant to medical treatment Endometrial premalignant lesions Cervical premalignant lesions	Myoma Uteri Adenomyozis Endometriozis AUB resistant to medical treatment Endometrial premalignant lesions Cervical premalignant lesions
Exclusion Criteria			
Indications	Myoma uteri Endometriosis Adenomyozis Endometrial premalignant lesions Cervical premalignant lesions	POP Endometriosis	POP
Obesity	Morbidly obese		

**Table 2:** Study groups and demographic characteristics.

All Patients (*n* = 280)	
Age (year) Mean ± SD	53.73 ± 8.41
**Hysterectomy Type, *n* (%)**	
TAH 1	29 (10.4)
TAH 2	55 (19.6)
TLH	88 (31.4)
VH	34 (12.2)
VH Mujas	74 (26.4)
**Indication, *n* (%)**	
Uterine fibroids	56 (20.0)
Adenomyosis	45 (6.1)
POP (pelvic organ prolapse)	75 (26.8)
Endometrial premalignant lesions	19 (6.8)
AUB resistant to medical treatment	59 (21.1)
Cervical premalignant lesions	14 (5.0)
Endometriosis	12 (4.2)
Mean uterine volume	510.24 ± 558.74 cm^3^
History of previous abdominal surgery	
None	80 (28.57)
Exist	200 (71.42)
Median (Min–Max)	1.0 (0.0–6.0)
**BMI, *n* (%)**	
Low	16 (5.7)
Normal	97 (34.6)
High	92 (32.9)
Obese	72 (25.7)
Morbid obese	3 (1.1)
**Education status *n* (%)**	
Primary	173 (61.8)
Middle/High school	93 (33.2)
University	14 (5.0)
**Parity, *n* (%)**	
Virgo	3 (1.0)
Nulliparous	7 (2.5)
Multipar	157 (56.1)
Grandmultipar	113 (40.4)
**Systemic disease, *n* (%)**	
None	186 (66.4)
Exist	94 (33.6)
**Habit, *n* (%)**	
None	252 (90.0)
Smoking	28 (10.0)

**Table 3:** Comparison of education, gravida-parity, systemic disease, and habits according to hysterectomy type.

	Hysterectomy Type					
	TAH 1 (*n* = 29)	TAH 2 (*n* = 55)	TLH (*n* = 88)	VH (*n* = 34)	VH Mujas (*n* = 74)	*p* *
	*n* (%)	*n* (%)	*n* (%)	*n* (%)	*n* (%)	
**Education**						
Primary	25 (86.2)	35 (63.6)	39 (44.3)	24 (70.6)	50 (67.6)	0.002
Middle/High school	4 (13.8)	18 (32.7)	41 (46.6)	8 (23.5)	22 (29.7)	
University	0 (0.0)	2 (3.6)	8 (9.1)	2 (5.9)	2 (2.7)	
**Parity**						
Virgo	2 (6.9)	0 (0.0)	0 (0.0)	1 (2.9)	0 (0.0)	0.008
Nulliparous	0 (0.0)	2 (3.6)	5 (5.7)	0 (0.0)	0 (0.0)	
Multipar	11 (37.9)	28 (50.9)	51 (58.0)	17 (50.0)	50 (67.6)	
Grandmultipar	16 (55.2)	25 (45.5)	32 (36.4)	16 (47.1)	24 (32.4)	
**Systemic Disease**						
None	11 (37.9)	37 (67.3)	63 (71.6)	21 (61.8)	54 (73.0)	0.010
Exist	18 (62.1)	18 (32.7)	25 (28.4)	13 (38.2)	20 (27.0)	
**Habit**						
None	25 (86.2)	45 (81.8)	83 (94.3)	33 (97.1)	66 (89.2)	0.081
Smoking	4 (13.8)	10 (18.2)	5 (5.7)	1 (2.9)	8 (10.8)	

* Chi square test.

**Table 4:** Comparison of parameters according to hysterectomy type.

	Hysterectomy Type					
	TAH 1 (*n* = 29)	TAH 2 (*n* = 55)	TLH (*n* = 88)	VH (*n* = 34)	VH Mujas (*n* = 74)	
	Mean ± SD Median (Min–Max)	Mean ± SD Median (Min–Max)	Mean ± SD Median (Min–Max)	Mean ± SD Median (Min–Max)	Mean ± SD Median (Min–Max)	*p*
**Age (year)**	53.21 ± 5.68	49.67 ± 5.49	52.85 ± 7.22	58.47 ± 8.27	55.81 ± 10.69	<0.001 ^a^
	53.0 (45.0–75.0)	50.0 (38.0–60.0)	52.0 (38.0–86.0)	57.0 (45.0–77.0)	55.0 (38.0–82.0)	
**Volume** (cm^3^)	1035.88 ± 1037.12	671.59 ± 617.63	439.04 ± 396.31	344.26 ± 289.88	345.25 ± 311.99	<0.001 ^a^
	756.0 (86.4–5040.0)	462.0 (84.0–3500.0)	333.0 (24.0–1417.5)	166.1 (54.0–910.0)	243.7 (54.0–1417.5)	
**Indication, *n* (%)**						
Myoma	14 (48.4)	22 (40.0)	20 (22.7)	0 (0.0)	0 (0.0)	<0.001 ^b^
Adenomyosis	4 (13.8)	14 (25.5)	27 (30.7)	0 (0.0)	0 (0.0)	
POP	0 (0.0)	0 (0.0)	0 (0.0)	24 (70.6)	51 (68.9)	
Endometrial premalignant lesions	5 (17.2)	4 (7.3)	10 (11.4)	0 (0.0)	0 (0.0)	
AUB resistant to medical treatment	5 (17.2)	7 (12.7)	14 (15.9)	10 (29.4)	23 (31.1)	
Cervical premalignant lesions	0 (0.0)	8 (14.5)	6 (6.8)	0 (0.0)	0 (0.0)	
Endometriosis	1 (3.4)	0 (0.0)	11 (12.5)	0 (0.0)	0 (0.0)	
History of previous abdominal surgery	2.0 (0.0–4.0)	2.0 (0.0–6.0)	1.0 (0.0–6.0)	1.0 (0.0–5.0)	1.0 (0.0–6.0)	0.001 ^a^
**BMI, *n* (%)**						
Low	2 (6.9)	0 (0.0)	1 (1.2)	6 (17.6)	7 (9.5)	<0.001 ^b^
Normal	5 (17.2)	30 (54.5)	25 (28.4)	11 (32.4)	26 (35.1)	
High	8 (27.6)	8 (14.5)	48 (54.5)	6 (17.6)	22 (29.7)	
Obese	14 (48.3)	14 (25.5)	14 (15.9)	11 (32.4)	19 (25.7)	
Morbidly obese	0 (0.0)	3 (5.5)	0 (0.0)	0 (0.0)	0 (0.0)	
Hospital stay duration	2.0 (2.0–7.0)	2.0 (2.0–2.0)	2.0 (1.0–5.0)	2.0 (1.0–5.0)	1.0 (1.0–2.0)	<0.001 ^a^
**Readmission to hospital, *n* (%)**						
No	23 (79.3)	54 (98.2)	87 (98.9)	34 (100.0)	72 (97.3)	0.002 ^b^
Yes	6 (20.7)	1 (1.8)	1 (1.1)	0 (0.0)	2 (2.7)	
**Pre-op Complication, *n* (%)**						
No	29 (100.0)	55 (100.0)	87 (98.9)	32 (94.1)	73 (98.6)	0.269 ^b^
Yes	0 (0.0)	0 (0.0)	1 (1.1)	2 (5.9)	1 (1.4)	
**Post-op Complication, *n* (%)**						
No	24 (82.8)	54 (98.2)	86 (97.7)	30 (88.2)	73 (98.6)	0.007 ^b^
Yes	5 (17.2)	1 (1.8)	2 (2.3)	4 (11.8)	1 (1.4)	
Time to complete hysterectomy	67.76 ± 12.79	43.18 ± 12.15	65.11 ± 23.69	51.03 ± 15.36	32.77 ± 7.22	<0.001 ^a^
	60.0 (50.0–90.0)	45.0 (20.0–90.0)	60.0 (30.0–120.0)	50.0 (25.0–90.0)	35.0 (15.0–50.0)	
**Sacrospinous Fixation, *n* (%)**						
None	29 (100.0)	55 (100.0)	88 (100.0)	13 (38.2)	44 (59.5)	<0.001 ^b^
Exist	0 (0.0)	0 (0.0)	0 (0.0)	21 (61.8)	30 (40.5)	
**Mc Coll Culdoplasty, *n* (%)**						
None	29 (100.0)	55 (100.0)	87 (98.9)	0 (0.0)	0 (0.0)	<0.001 ^b^
Exist	0 (0.0)	0 (0.0)	1 (1.1)	34 (100.0)	74 (100.0)	
**Shortening of the Uterosacral Ligament, *n* (%)**						
None	0 (0.0)	0 (0.0)	0 (0.0)	34 (100.0)	74 (100.0)	<0.001 ^b^
Exist	29 (100.0)	55 (100.0)	88 (100.0)	0 (0.0)	0 (0.0)	
**1-Month Follow-up evaluation of satisfaction with the results of the surgery, *n* (%)**						
Not Sure	2 (7.1)	0 (0.0)	1 (1.2)	0 (0.0)	0 (0.0)	<0.001 ^b^
Satisfied	19 (67.9)	6 (10.9)	12 (13.6)	5 (14.7)	3 (4.1)	
Very Satisfied	7 (25.0)	49 (89.1)	75 (85.2)	29 (85.3)	71 (95.9)	
**Would you recommend your surgical method to others? *n* (%)**						
No, I would not	1 (3.6)	0 (0.0)	0 (0.0)	0 (0.0)	0 (0.0)	<0.001 ^b^
I am not sure	2 (7.1)	2 (3.6)	7 (8.0)	0 (0.0)	0 (0.0)	
Yes, I would	22 (78.6)	3 (5.5)	6 (6.8)	0 (0.0)	4 (5.4)	
Yes, I highly recommend	3 (10.7)	50 (90.9)	75 (85.2)	34 (100.0)	70 (94.6)	
**When did you return to your pre-operative daily routine? *n* (%)**						
First 3 days	0 (0.0)	0 (0.0)	30 (34.1)	25 (73.5)	64 (86.5)	<0.001 ^b^
First week	3 (10.3)	33 (61.1)	40 (45.5)	7 (20.6)	10 (13.5)	
First 15 days	18 (62.2)	11 (20.4)	16 (18.1)	2 (5.9)	0 (0.0)	
First month	7 (24.1)	9 (16.6)	2 (2.3)	0 (0.0)	0 (0.0)	
1 month later	1 (3.4)	1 (1.9)	0 (0.0)	0 (0.0)	0 (0.0)	

^a^: Kruskal–Wallis Test, ^b^: Chi square test.

**Table 5:** Comparison of HG parameters between groups and time.

	TAH 1		TAH 2		TLH		VH		VH Mujas		
	Mean ± SD	Median (Min–Max)	Mean ± SD	Median (Min–Max)	Mean ± SD	Median (Min–Max)	Mean ± SD	Median (Min–Max)	Mean ± SD	Median (Min–Max)	*p* (Group)
**HG**											
Pre-op	12.76 ± 1.12	12.0 (11–15)	12.60 ± 1.06	12.0 (11–15)	12.42 ± 1.15	12.0 (11–16)	12.65 ± 1.35	12.0 (11–16)	12.85 ± 1.49	13.0 (11–16)	0.403 ^a^
Post-op	9.72 ± 1.58	9.0 (7–14)	11.62 ± 1.39	12.0 (9–14)	10.98 ± 1.26	11.0 (8–15)	11.38 ± 1.39	11.0 (9–15)	11.88 ± 1.39	12.0 (9–15)	<0.001 ^a^
*p* (time)	<0.001 ^b^		<0.001 ^b^		<0.001 ^b^		<0.001 ^b^		<0.001 ^b^		

^a^: Kruskal–Wallis Testi, ^b^: Wilcoxon signed rank test.

**Table 6:** Comparison of VAS, FSFI, Beck Anxiety Scale, and SF36 parameters by groups and times.

	TAH 1		TAH 2		TLH		VH		VH Mujas		
	Mean ± SD	Median (Min–Max)	Mean ± SD	Median (Min–Max)	Mean ± SD	Median (Min–Max)	Mean ± SD	Median (Min–Max)	Mean ± SD	Median (Min–Max)	*p* (Group)
**VAS**											
Pre-op	2.38 ± 1.61	3.0 (0–6)	1.42 ± 2.18	0.0 (0–6)	2.95 ± 2.54	3.0 (0–8)	0.00 ± 0.00	0.0 (0–0)	0.03 ± 0.16	0.0 (0–1.0)	<0.001 ^a^
1st Day	6.86 ± 1.68	7.0 (4–9)	4.35 ± 1.66	4.0 (2–10)	4.94 ± 2.11	5.0 (2–10)	3.06 ± 0.74	3.0 (2–4)	1.80 ± 0.64	2.0 (1–3)	<0.001 ^a^
10th Day	3.38 ± 0.98	4.0 (2–5)	1.20 ± 1.24	1.0 (0–5)	1.23 ± 1.27	1.0 (0–5)	0.82 ± 0.90	1.0 (0–3)	0.24 ± 0.49	0.0 (0–2)	<0.001 ^a^
*p* (Time)	<0.001 ^c^		<0.001 ^c^		<0.001 ^c^		<0.001 ^c^		<0.001 ^c^		
**FSFI**											
Pre-op	21.71 ± 3.21	20.4 (16.7–26.2)	21.91 ± 2.85	21.8 (16.7–27.0)	21.94 ± 3.64	21.4 (16.7–30.1)	19.93 ± 3.55	19.3 (14.8–26.3)	19.28 ± 3.27	18.9 (14.8–27.1)	<0.001 ^a^
Post-op	24.77 ± 3.12	25.7 (18.5–28.6)	24.15 ± 3.14	24.7 (18.8–30.1)	25.67 ± 4.03	25.9 (16.7–35.5)	25.97 ± 3.16	26.2 (19.1–30.3)	27.70 ± 4.22	27.8 (14.8–35.5)	<0.001 ^a^
*p* (Time)	<0.001 ^b^		<0.001 ^b^		<0.001 ^b^		<0.001 ^b^		<0.001 ^b^		
**Beck Anxiety**											
1st interview	26.29 ± 17.29	19.0 (9.0–56.0)	19.80 ± 10.79	16.0 (6.0–48.0)	24.70 ± 13.99	22.0 (2.0–52.0)	30.24 ± 15.26	26.0 (10.0–58.0)	23.55 ± 11.68	25.0 (6.0–60.0)	0.015 ^a^
2nd interview	52.11 ± 7.19	54.5 (38.0–61.0)	36.11 ± 16.96	42.0 (4.0–60.0)	27.28 ± 14.66	26.0 (2.0–60.0)	24.94 ± 11.19	25.0 (8.0–42.0)	12.42 ± 5.49	12.0 (4.0–26.0)	<0.001 ^a^
*p* (Time)	<0.001 ^b^		<0.001 ^b^		<0.001 ^b^		0.005 ^b^		<0.001 ^b^		
**SF36 Physical Function**											
Pre-op	70.69 ± 24.26	65.0 (15–100)	76.64 ± 25.35	85.0 (10–100)	65.46 ± 21.03	65.0 (10–100)	54.85 ± 19.59	55.0 (10–95)	49.39 ± 20.59	55.0 (10–95)	<0.001 ^a^
Post-op	77.24 ± 19.44	70.0 (25–100)	82.36 ± 19.90	90.0 (35–100)	80.06 ± 15.11	80.0 (35–100)	85.29 ± 11.87	85.0 (55–100)	87.09 ± 10.98	85.0 (60–100)	0.020 ^a^
*p* (Time)	0.001 ^b^		<0.001 ^b^		<0.001 ^b^		<0.001 ^b^		<0.001 ^b^		
**SF36 Physical Role Difficulty**											
Pre	56.03 ± 44.15	50.0 (0–100)	63.18 ± 37.84	75.0 (0–100)	40.92 ± 34.30	50.0 (0–100)	40.44 ± 35.89	25.0 (0–100)	30.41 ± 27.22	25.0 (0–75)	<0.001 ^a^
Post	62.93 ± 35.11	50.0 (0–100)	75.91 ± 28.45	75.0 (0–100)	70.40 ± 26.01	75.0 (25–100)	87.50 ± 17.68	100.0 (50–100)	82.43 ± 20.58	87.5 (25–100)	0.001 ^a^
*p* (time)	0.033 ^b^		<0.001 ^b^		<0.001 ^b^		<0.001 ^b^		<0.001 ^b^		
**SF36 Emotional Role Difficulty**											
Pre-op	50.57 ± 46.82	33.3 (0–100)	58.77 ± 41.55	66.6 (0–100)	51.71 ± 42.15	66.6 (0–100)	28.41 ± 31.91	33.3 (0–100)	37.37 ± 36.98	33.3 (0–100)	0.005 ^a^
Post-op	57.45 ± 39.74	33.3 (0–100)	75.12 ± 31.58	100.0 (0–100)	72.01 ± 30.03	66.6 (0–100)	84.28 ± 18.80	100.0 (33–100)	88.72 ± 16.83	100.0 (33–100)	<0.001 ^a^
*p* (Time)	0.083 ^b^		0.004 ^b^		<0.001 ^b^		<0.001 ^b^		<0.001 ^b^		
**SF36 Energy/Vitality**											
Pre-op	47.07 ± 20.55	40.0 (15–90)	49.09 ± 20.21	55.0 (5–90)	44.94 ± 17.68	40.0 (10–80)	43.68 ± 14.94	45.0 (10–75)	43.92 ± 17.85	50.0 (10–90)	0.633 ^a^
Post-op	56.03 ± 18.49	55.0 (30–90)	60.64 ± 18.05	60.0 (10–90)	66.55 ± 13.82	65.0 (40–90)	71.76 ± 14.35	75.0 (40–90)	76.74 ± 13.45	80.0 (9–90)	<0.001 ^a^
*p* (Time)	<0.001 ^b^		<0.001 ^b^		<0.001 ^b^		<0.001 ^b^		<0.001 ^b^		
**SF36 Spiritual Health**											
Pre-op	50.76 ± 22.65	44.0 (20–92)	59.20 ± 18.42	60.0 (4–88)	54.57 ± 17.56	56.0 (4–88)	50.71 ± 20.34	44.0 (20–88)	50.00 ± 16.44	52.0 (4–80)	0.015 ^a^
Post-op	62.03 ± 21.51	64.0 (32–92)	67.64 ± 14.63	64.0 (40–92)	70.57 ± 13.04	72.0 (44–92)	80.47 ± 9.33	80.0 (60–92)	82.70 ± 8.76	84.0 (60–92)	<0.001 ^a^
*p* (Time)	<0.001 ^b^		<0.001 ^b^		<0.001 ^b^		<0.001 ^b^		<0.001 ^b^		
**SF36 Social Functionality**											
Pre-op	59.22 ± 28.12	62.5 (0–100)	67.77 ± 26.77	75.0 (12–100)	58.42 ± 25.49	62.5 (0–100)	46.10 ± 28.32	50.0 (0–100)	55.68 ± 27.99	62.5 (0–100)	0.018 ^a^
Post-op	76.47 ± 21.06	75.0 (25–100)	81.45 ± 19.65	87.5 (25–100)	77.79 ± 20.04	75.0 (25–100)	77.50 ± 15.07	75.0 (37–100)	85.34 ± 13.88	88.7 (37–100)	0.085 ^a^
*p* (Time)	<0.001 ^b^		<0.001 ^b^		<0.001 ^b^		<0.001 ^b^		<0.001 ^b^		
**SF36 Pain**											
Pre-op	50.52 ± 28.78	45.0 (12–100)	56.18 ± 24.99	57.5 (10–100)	69.19 ± 27.49	67.5 (10–100)	94.48 ± 12.02	100.0 (42–100)	97.29 ± 6.26	100.0 (80–100)	<0.001 ^a^
Post-op	62.41 ± 23.57	57.5 (32–100)	70.36 ± 19.25	77.5 (22–100)	96.72 ± 7.51	100.0 (67–100)	100.00 ± 0.00	100.0 (100–100)	100.00 ± 0.00	100.0 (100–100)	<0.001 ^a^
*p* (Time)	<0.001 ^b^		<0.001 ^b^		<0.001 ^b^		0.007 ^b^		0.001 ^b^		
**SF36 General Health Perception**											
Pre-op	50.83 ± 22.88	55.0 (20–90)	52.40 ± 17.87	50.0 (15–90)	50.47 ± 15.05	50.0 (20–80)	53.44 ± 15.00	51.0 (30–85)	43.64 ± 15.97	42.2 (15–85)	0.014 ^a^
Post-op	61.55 ± 16.86	60.0 (35–90)	67.00 ± 15.41	70.0 (35–90)	68.13 ± 12.62	70.0 (40–90)	78.68 ± 13.04	85.0 (35–90)	74.53 ± 12.58	75.0 (45–90)	<0.001 ^a^
*p* (Time)	<0.001 ^b^		<0.001 ^b^		<0.001 ^b^		<0.001 ^b^		<0.001 ^b^		

^a^: Kruskal–Wallis test, ^b^: Wilcoxon signed rank test, ^c^: Friedman test.

**Table 7:** Inter-group comparisons of VAS, FSFI, Beck Anxiety Scale, and SF36 values in different types of hysterectomy: pairwise analyses.

Groups—VAS Pre-Operative	*p*	Groups—VAS 1st Day	*p*
TAH 2—TLH	0.001	TAH MUJAS—TLH	1.000
TAH 2—TAH	0.029	TAH MUJAS—TAH	0.001
TAH 2—VTH Mujas	0.002	TAH MUJAS—VTH MUJAS	<0.001
TAH 2—VTH	0.016	TAH MUJAS—VTH	0.072
TLH—TAH	1.000	TLH—TAH	0.008
TLH—VTH Mujas	<0.001	TLH—VTH MUJAS	<0.001
TLH—VTH	<0.001	TLH—VTH	0.001
TAH—VTH Mujas	<0.001	TAH—VTH MUJAS	<0.001
TAH—VTH	<0.001	TAH—VTH	<0.001
VTH Mujas—VTH	1.000	VTH MUJAS—VTH	0.001
**Groups—VAS 10th Day**	***p***	**Groups—Pre-operative FSFI**	***p***
TAH 2—TLH	1.000	TAH 2—TLH	1.000
TAH 2—TAH	<0.001	TAH 2—TAH	1.000
TAH 2—VTH Mujas	<0.001	TAH 2—VTH Mujas	<0.001
TAH 2—VTH	1.000	TAH 2—VTH	0.071
TLH—TAH	<0.001	TLH—TAH	1.000
TLH—VTH Mujas	<0.001	TLH—VTH Mujas	<0.001
TLH—VTH	1.000	TLH—VTH	0.124
TAH—VTH Mujas	<0.001	TAH—VTH Mujas	0.011
TAH—VTH	<0.001	TAH—VTH	0.380
VTH Mujas —VTH	0.081	VTH Mujas—VTH	1.000
**Groups—Post-operative FSFI**	***p***	**Groups—Pre-operative Beck Anxiety**	***p***
TAH 2—TLH	0.114	TAH 2—TLH	0.313
TAH 2—TAH	1.000	TAH 2—TAH	1.000
TAH 2—VTH Mujas	<0.001	TAH 2—VTH Mujas	0.394
TAH 2—VTH	0.134	TAH 2—VTH	0.006
TLH—TAH	1.000	TLH—TAH	1.000
TLH—VTH Mujas	0.006	TLH—VTH Mujas	1.000
TLH—VTH	1.000	TLH—VTH	0.587
TAH—VTH Mujas	0.008	TAH—VTH Mujas	1.000
TAH—VTH	1.000	TAH—VTH	0.798
VTH Mujas —VTH	0.353	VTH Mujas—VTH	0.631
**Groups—Post-operative Beck Anxiety**	***p***	**Groups—Pre-operative** **SF-36 Physical Functioning**	***p***
TAH 2—TLH	0.233	TAH 2—TLH	0.024
TAH 2—TAH	0.003	TAH 2—TAH	1.000
TAH 2—VTH MUJAS	<0.001	TAH 2—VTH MUJAS	<0.001
TAH 2—VTH	0.194	TAH 2—VTH	<0.001
TLH—TAH	<0.001	TLH—TAH	1.000
TLH—VTH Mujas	<0.001	TLH—VTH Mujas	0.001
TLH—VTH	1.000	TLH—VTH	0.242
TAH—VTH Mujas	<0.001	TAH—VTH Mujas	0.001
TAH—VTH	<0.001	TAH—VTH	0.047
VTH Mujas —VTH	<0.001	VTH Mujas—VTH	1.000
**Groups—Post-operative SF-36 Physical Functioning**	***p***	**Groups—Pre-operative SF36 Role Limitations Due To Physical Health**	***p***
TAH 2—TLH	0.718	TAH MUJAS—TLH	0.006
TAH 2—TAH	0.596	TAH MUJAS—TAH	1.000
TAH 2—VTH Mujas	1.000	TAH MUJAS—VTH MUJAS	<0.001
TAH 2—VTH	1.000	TAH MUJAS—VTH	0.058
TLH—TAH	1.000	TLH—TAH	0.719
TLH—VTH Mujas	0.006	TLH—VTH MUJAS	0.802
TLH—VTH	1.000	TLH—VTH	1.000
TAH—VTH Mujas	0.011	TAH—VTH MUJAS	0.025
TAH—VTH	0.913	TAH—VTH	1.000
VTH Mujas—VTH	1.000	VTH MUJAS—VTH	1.000
**Groups—Post-operative SF36 Role Limitations Due To Physical Health**	***p***	**Groups—Pre-operative SF-36 Role Limitations Due To Emotional Problems**	***p***
TAH 2—TLH	1.000	TAH 2—TLH	1.000
TAH 2—TAH	0.919	TAH 2—TAH	1.000
TAH 2—VTH Mujas	1.000	TAH 2—VTH Mujas	0.061
TAH 2—VTH	0.725	TAH 2—VTH	0.014
TLH—TAH	1.000	TLH—TAH	1.000
TLH—VTH Mujas	0.047	TLH—VTH Mujas	0.367
TLH—VTH	0.011	TLH—VTH	0.075
TAH—VTH Mujas	0.100	TAH—VTH Mujas	1.000
TAH—VTH	0.021	TAH—VTH	0.451
VTH Mujas—VTH	1.000	VTH Mujas—VTH	1.000
**Groups—Post-operative SF-36 Role Limitations Due To Emotional Problems**	***p***	**Groups—Post-operative SF-36 Energy/Vitality**	***p***
TAH 2—TLH	1.000	TAH 2—TLH	1.000
TAH 2—TAH	0.442	TAH 2—TAH	1.000
TAH 2—VTH Mujas	0.206	TAH 2—VTH Mujas	<0.001
TAH 2—VTH	1.000	TAH 2—VTH	0.051
TLH—TAH	1.000	TLH—TAH	0.256
TLH—VTH Mujas	0.006	TLH—VTH Mujas	<0.001
TLH—VTH	0.821	TLH—VTH	0.628
TAH—VTH Mujas	0.001	TAH—VTH Mujas	<0.001
TAH—VTH	0.068	TAH—VTH	0.007
VTH Mujas—VTH	1.000	VTH Mujas—VTH	0.935
**Groups—Pre-operative SF36 Emotional Well-Being**	***p***	**Groups—Post-operative SF36 Emotional Well-Being**	***p***
TAH 2—TLH	1.000	TAH 2—TLH	1.000
TAH 2—TAH	0.016	TAH 2—TAH	1.000
TAH 2—VTH Mujas	0.005	TAH 2—VTH Mujas	<0.001
TAH 2—VTH	0.007	TAH 2—VTH	0.001
TLH—TAH	1.000	TLH—TAH	1.000
TLH—VTH Mujas	0.906	TLH—VTH Mujas	<0.001
TLH—VTH	1.000	TLH—VTH	0.005
TAH—VTH Mujas	1.000	TAH—VTH Mujas	<0.001
TAH—VTH	1.000	TAH—VTH	0.002
VTH Mujas—VTH	1.000	VTH Mujas—VTH	1.000
**Groups—Pre-operative SF36 Social Functioning**	***p***	**Groups—Pre-operative SF36 Pain**	***p***
TAH 2—TLH	0.663	TAH 2—TLH	0.024
TAH 2—TAH	1.000	TAH 2—TAH	1.000
TAH 2—VTH Mujas	0.252	TAH 2—VTH Mujas	<0.001
TAH 2—VTH	0.008	TAH 2—VTH	<0.001
TLH—TAH	1.000	TLH—TAH	0.033
TLH—VTH Mujas	1.000	TLH—VTH Mujas	<0.001
TLH—VTH	0.401	TLH—VTH	<0.001
TAH—VTH Mujas	1.000	TAH—VTH Mujas	<0.001
TAH—VTH	0.803	TAH—VTH	<0.001
VTH Mujas—VTH	1.000	VTH Mujas—VTH	1.000
**Groups—Post-operative SF36 Pain**	***p***	**Groups—Pre-operative General Health**	***p***
TAH 2—TLH	<0.001	TAH 2—TLH	1.000
TAH 2—TAH	1.000	TAH 2—TAH	1.000
TAH 2—VTH Mujas	<0.001	TAH 2—VTH Mujas	0.038
TAH 2—VTH	<0.001	TAH 2—VTH	1.000
TLH—TAH	<0.001	TLH—TAH	1.000
TLH—VTH Mujas	0.442	TLH—VTH Mujas	0.089
TLH—VTH	1.000	TLH—VTH	1.000
TAH—VTH Mujas	<0.001	TAH—VTH Mujas	0.624
TAH—VTH	<0.001	TAH—VTH	1.000
VTH Mujas—VTH	1.000	VTH Mujas—VTH	0.069
**Groups—Post-operative General Health**	***p***		
TAH 2—TLH	1.000		
TAH 2—TAH	1.000		
TAH 2—VTH Mujas	0.046		
TAH 2—VTH	0.001		
TLH—TAH	1.000		
TLH—VTH Mujas	0.023		
TLH—VTH	<0.001		
TAH—VTH Mujas	0.002		
TAH—VTH	<0.001		
VTH Mujas—VTH	0.968		

## Data Availability

The data generated and analyzed during the study are available from the corresponding author. They are not available publicly.
